# Maternal and fetal predictors of anthropometry in the first year of life in offspring of women with GDM

**DOI:** 10.3389/fendo.2023.1144195

**Published:** 2023-03-28

**Authors:** Maria-Christina Antoniou, Dan Yedu Quansah, Suzanne Mühlberg, Leah Gilbert, Amar Arhab, Sybille Schenk, Alain Lacroix, Bobby Stuijfzand, Antje Horsch, Jardena Jacqueline Puder

**Affiliations:** ^1^ Unit of Pediatric Endocrinology and Diabetology, Pediatric Service, Woman-Mother-Child Department, Lausanne University Hospital, Lausanne, Switzerland; ^2^ Obstetric Service, Woman-Mother-Child Department, Lausanne University Hospital, Lausanne, Switzerland; ^3^ Faculty of Biology and Medicine, University of Lausanne, Lausanne, Switzerland; ^4^ Institute of Higher Education and Research in Healthcare (IUFRS), University of Lausanne, Lausanne, Switzerland; ^5^ Neonatology Service, Woman-Mother-Child Department, Lausanne University Hospital, Lausanne, Switzerland

**Keywords:** gestational diabetes, cord blood, offspring anthropometry, fetal metabolism, maternal metabolism

## Abstract

**Introduction:**

Gestational Diabetes Mellitus (GDM) carries an increased risk for adverse perinatal and longer-term cardiometabolic consequences in offspring. This study evaluated the utility of maternal anthropometric, metabolic and fetal (cord blood) parameters to predict offspring anthropometry up to 1 year in pregnancies with GDM.

**Materials and methods:**

In this prospective analysis of the *MySweetheart* study, we included 193/211 women with GDM that were followed up to 1 year postpartum. Maternal predictors included anthropometric (pre-pregnancy BMI, gestational weight gain (GWG), weight and fat mass at the 1^st^ GDM visit), and metabolic parameters (fasting insulin and glucose, Homeostatic Model Assessment for Insulin Resistance (HOMA-IR), Quantitative insulin-sensitivity check index (QUICKI), HbA1c, triglycerides, and high-density lipoprotein (HDL) at the 1^st^ visit and HbA1c at the end of pregnancy). Fetal predictors (N=46) comprised cord blood glucose and insulin, C-Peptide, HOMA-IR, triglycerides and HDL. Offspring outcomes were anthropometry at birth (weight/weight z-score, BMI, small and large for gestational age (SGA,LGA)), 6-8 weeks and 1 year (weight z-score, BMI/BMI z-score, and the sum of 4 skinfolds).

**Results:**

In multivariate analyses, birth anthropometry (weight, weight z-score, BMI and/or LGA), was positively associated with cord blood HDL and HbA1c at the 1^st^ GDM visit, and negatively with maternal QUICKI and HDL at the 1^st^ GDM visit (all p ≤ 0.045). At 6-8 weeks, offspring BMI was positively associated with GWG and cord blood insulin, whereas the sum of skinfolds was negatively associated with HDL at the 1^st^ GDM visit (all p ≤0.023). At 1 year, weight z-score, BMI, BMI z-score, and/or the sum of skinfolds were positively associated with pre-pregnancy BMI, maternal weight, and fat mass at the 1^st^ GDM visit and 3^rd^ trimester HbA1c (all p ≤ 0.043). BMI z-score and/or the sum of skinfolds were negatively associated with cord blood C-peptide, insulin and HOMA-IR (all p ≤0.041).

**Discussion:**

Maternal anthropometric, metabolic, and fetal metabolic parameters independently affected offspring anthropometry during the 1^st^ year of life in an age-dependent manner. These results show the complexity of pathophysiological mechanism for the developing offspring and could represent a base for future personalized follow-up of women with GDM and their offspring.

## 1 Introduction

Gestational Diabetes Mellitus (GDM) is defined as diabetes first diagnosed during the second or third trimester of pregnancy, not fulfilling the criteria of pre-existing diabetes (1). The prevalence of GDM varies significantly worldwide, ranging from 1% to > 30% and is ~ 11% in Switzerland (2). It has been suggested that the intrauterine environment of GDM may affect fetal programming and future health in offspring of mothers with GDM (3–5). GDM carries an increased risk for adverse perinatal outcomes, such as large for gestational age (LGA) and increased adiposity, birth trauma, respiratory distress syndrome, jaundice, hypoglycemia, and admission to the neonatal intensive care unit (6–8). The impact of GDM on offspring anthropometry is present at birth and in later childhood (4, 8, 9), but data during the 1^st^ year of life in this population are lacking. A higher body mass index (BMI) as well as an increased risk for overweight and obesity during childhood has been found in GDM-exposed offspring in most studies (4, 10, 11). In the HAPO study, GDM was positively associated with childhood overweight or obesity at a mean age of 11.4 years and this was mediated by the maternal BMI during pregnancy (9). These findings are in agreement with the KiGGS study (12). Data on cardiometabolic consequences of GDM in the offspring have been in part inconsistent. They might include elevated blood pressure (13) and a higher risk for dyslipidemia (14). They have a higher risk of impaired glucose metabolism (15, 16) during childhood and adolescence and an increased risk for obesity, insulin resistance, metabolic syndrome, prediabetes, and type 2 diabetes during early adulthood (17, 18).

In women with GDM, maternal anthropometric (pre-pregnancy BMI, GWG) and metabolic parameters (glucose values during oGTT, HbA1c, C-peptide and lipids) during the 2^nd^ and 3^rd^ trimester of pregnancy, have been associated with neonatal anthropometry including birth weight, BMI, macrosomia, large and small for gestational age (LGA, SGA) (19–23). Although data on the impact of cord blood metabolic parameters on neonatal anthropometry and adiposity are available in the general population (24, 25), they are limited in the GDM population. In pregnancies with GDM, cord blood insulin, C-peptide, and glucose values have been associated with weight, BMI, the sum of skinfolds and fat mass at birth, whereas the impact of fetal lipid metabolism remains controversial (26–29). Previous studies have assessed these associations only in older offspring, i.e., during childhood and adolescence (9, 11, 12, 30). The impact of maternal and fetal metabolism on infant anthropometry at different time points during the 1^st^ year of life has not been studied in the GDM population and data in the general population are scarce. This might shed light on different pathophysiological processes regarding developmental aspects of metabolic health.

The aim of this study was to evaluate the utility of maternal anthropometric and metabolic, as well as fetal (cord blood) parameters as predictors of infant anthropometric and adiposity outcomes at different time points during the 1^st^ year of life in pregnancies with GDM.

## 2 Materials and methods

### 2.1 Study design and follow-up

This study is a secondary analysis of the *MySweetheart trial*, a randomized-controlled intervention trial of 211 women with GDM and their offspring (Clinicaltrials.gov NCT02890693) (31). They were followed during pregnancy up to one year postpartum between 2016 and 2021, in the Diabetes and Pregnancy Unit in the Lausanne University Hospital, Switzerland. The intervention consisted of a multidimensional interdisciplinary lifestyle and psychosocial intervention in women with GDM and their offspring compared with an active lifestyle and guidelines-based usual care. The allocation ratio was 1:1 using a block randomization method (blocks of 4) after stratification. Details of the study protocol have been described elsewhere (31).

#### 2.1.1 Participant recruitment and consent

Women ≥18 years diagnosed with GDM between 24 and 32 weeks of gestational age (GA), who understood French or English, and consented to participate were included in *MySweetheart trial*. Women on strict bed rest, with severe mental disorder and pre-existing diabetes were excluded from the study. Signed informed consent was obtained from all participating women. The study was conducted in accordance with the guidelines of the declaration of Helsinki, and good clinical practice. The Human Research Ethics Committee of the Canton de Vaud approved the study protocol (study number 2016-00745).

#### 2.1.2 Diagnosis of gestational diabetes

GDM was diagnosed according to the International Association of Diabetes and Pregnancy Study Group (IADPSG Criteria). GDM was confirmed if fasting blood glucose was ≥5.1 mmol/l and/or 1-h blood glucose was ≥10.0 mmol/l and/or 2-h blood glucose was ≥8.5 mmol/l, following a 75 g oGTT (32). Included women were randomized to the control or intervention group after the baseline visit and signing of an informed consent.

#### 2.1.3 Follow-up

##### 2.1.3.1 Control group

Women randomized to usual care (N=106 women with GDM) received a very active guideline-based treatment-as-usual clinical follow-up based on the American Diabetes Association and on the Endocrine Society guidelines for the management of GDM (33, 34). They had regular appointments every 1-3 weeks with a medical doctor, a diabetes-specialist nurse and/or a dietician after the GDM diagnosis. During the 1^st^ visit, women were counselled on GDM and taught how to perform self-monitoring of blood glucose control 4 times during the day (fasting and 2 hours post-prandial). They were also advised on gestational weight gain (GWG) based on the Institute of Medicine (IOM) 2009 recommendations (35, 36). Patients had one appointment with a dietician for a personalized dietary counselling,and were encouraged to increase physical activity according to the Endocrine Society Guidelines (33). If glucose values remained above targets two or more times during a 1 to 2-week period (fasting glucose >5.3 mmol/l, 1-h postprandial glucose >8 mmol/l and/or 2-h postprandial glucose >7 mmol/l) despite lifestyle changes, insulin treatment (or very rarely metformin) was introduced depending on patient’s glucose values and preference. After delivery, capillary glucose measures and glucose-lowering treatments were stopped and women saw a physician and a dietician at the 6-8 weeks postpartum after an oGTT test to discuss further management and receive lifestyle counselling.

##### 2.1.3.2 Intervention group

Women randomized to the intervention group (N=105 women) received a multidimensional, interdisciplinary lifestyle and psychosocial intervention on top of the usual care. The focus was on eating behavior and a balanced food intake as well as physical activity and breastfeeding. The intervention also included a psychosocial component including the assessment of depression during and after pregnancy. Throughout the period of pregnancy and up to 1 year postpartum, patients were supported by a lifestyle coach (see (31) for more details).

##### 2.1.3.3 Visits

Women were evaluated at different moments during the study, i.e., at the 1^st^ GDM visit at 24-32 weeks (baseline; visit 1), at birth (visit 2), at 6-8 weeks postpartum (visit 3), and at 1 year postpartum (visit 4). Offspring were evaluated at birth, 6-8 weeks and at 1 year. At each visit, several measures were assessed. In the following section, we only mention measures that were analyzed in this present study.

Visit 1-1^st^ GDM visit: At 24-32 weeks of GA information on maternal socio-demographic characteristics were collected and maternal anthropometric parameters and fasting metabolic biomarkers were measured.

Visit 2- Birth: Immediately after childbirth, blood was drawn from the umbilical cord to measure laboratory biomarkers. Offspring anthropometric parameters were obtained from the hospital birth record.

Visit 3- 6-8 Weeks: At 6-8 weeks of life, offspring’s anthropometric measures including weight, length, BMI and skinfold measures were obtained.

Visit 4- 1 year: At this visit, offspring’s anthropometric measures including weight, BMI, skinfold measures were collected.

### 2.2 Maternal and offspring parameters

#### 2.2.1 Maternal sociodemographic and anthropometric parameters

Maternal socio-demographic parameters, including age, ethnicity, and parity were collected during a structured face-to-face interview at the 1^st^ GDM clinical visit. Ethnicity was classified in Low (Europe, North America) and High Risk (Asia, Central and South America, Africa, Oceania) ethnic groups (37). Pre-pregnancy BMI was calculated based on self-reported pre-pregnancy weight or retrieved from medical charts and measured height on the 1^st^ visit at the GDM clinic1. Weight was measured at the 1^st^ GDM visit to the nearest 0.1 kg in women wearing light clothes and no shoes with an electronic Seca ^®^ scale. Height was measured at the 1^st^ GDM visit to the nearest 0.1 cm with a regularly calibrated Seca ^®^ height scale. GWG was determined as the difference between the weight at the end of pregnancy and pre-pregnancy weight. At the 1^st^ GDM visit, Bioelectrical Impedance Analysis (BIA) was performed (Akern BIA 101) to estimate fat free mass (FFM) using the Kyle equation (38), and fat mass was calculated using the formula: **Fat Mass = Weight – FFM**. Maternal medical treatment for GDM was classified in 2 categories (no treatment, treatment with insulin and/or very rarely metformin).

#### 2.2.2 Offspring anthropometric parameters

Birth growth parameters such as weight (g) and length (cm) were documented at birth; percentiles and z-scores for each of the above-mentioned parameters were calculated using the Intergrowth 21^st^ newborn size application tool (39) and BMI was calculated. LGA was defined as birth weight >90^th^ percentile and SGA as birth weight <10^th^ percentile for sex and gestational age. Gestational age was calculated according to the date of the last menstruation, or as assessed by the fetal ultrasound in the cases where gestational age was corrected during the early *in-utero* ultrasound evaluation. Neonatal data were obtained from patient medical chart for all newborns born in the Lausanne University Hospital. In the cases where delivery took place in another hospital or clinic, anthropometric parameters at birth were provided by the respective hospital.

At the 6-8 weeks and 1 year visits, offspring weight (kg) and length (cm) were measured. Weight was measured to the closest 0.1 kg, with a calibrated scale (Seca ^®^ model 336). Babies were weighed without any clothes or in nappy. If the weight was measured with the nappy, the respective weight of the nappy was subtracted. Length was measured to the closest 0.1 cm with the same scale (Seca ^®^ model 336) and BMI was calculated. Z-scores for weight, length and BMI were calculated using the using the WHO Anthro Survey Analyser tool -Offline version (40).

Skinfold thickness was measured to the nearest 0.1mm at 4 anatomical sites (biceps, triceps, subscapular, and iliac) using a Harpenden Skinfold Caliper. Skinfolds were measured three times at each anatomical site and mean value of each skinfold measure was used to calculate the sum of the 4 skinfolds.

#### 2.2.3 Maternal and fetal (cord blood) metabolic parameters

At the 1^st^ GDM visit, maternal metabolic health parameters, including fasting glucose, insulin, HbA1c, high-density lipoprotein (HDL), and triglycerides were measured. Maternal HbA1c also were measured at the end of pregnancy (last visit before delivery). At birth, glucose, insulin, C-peptide, high-density lipoprotein (HDL), and triglycerides were measured in the cord blood. The Homeostatic Model Assessment for Insulin Resistance (HOMA-IR) was calculated using the formula (Fasting insulin in mIU/L x Fasting glucose in mmol/l)/22.5 (41). The Quantitative insulin-sensitivity check index (QUICKI) was calculated using the formula QUICKI = 1/[log(Fasting insulin in mIU/L) + log(Fasting glucose in mg/dl)] (42).

#### 2.2.4 Laboratory methods

Plasma glucose was measured using a Hexokinase/Glucose-6-Phosphat-Dehydrogenase (HK/G6P-DH) assay. Insulin and C-peptide were measured with an electrochemiluminescence Immunoassay. HbA1c was measured using a chemical photometric method (conjugation with boronate; Afinion^®^). HDL cholesterol and triglycerides were measured with an enzymatic colorimetric method (CHOD-PAP and GPO-PAP respectively).

#### 2.2.5 Predictors and outcomes

Maternal *anthropometric* parameters comprised of pre-pregnancy BMI, 1^st^ GDM visit weight and fat mass (BIA) and GWG. Maternal *metabolic* parameters included fasting glucose, insulin, HOMA-IR, QUICKI, HDL, triglycerides as well as HbA1c at the 1^st^ and last GDM visit. Cord blood *metabolic* parameters included glucose, insulin, C-peptide, HOMA-IR, HDL, and triglycerides. Outcomes comprised offspring anthropometric parameters at birth, 6-8 weeks and 1 year. More precisely, birth outcomes included weight z-score, BMI, LGA, and SGA, and outcomes at 6-8 weeks and 1 year, weight z-score, BMI, BMI z-score, and the sum of 4 skinfolds.

### 2.3 Statistical analysis

All data were analysed using Stata/SE 16.0 (StataCorp LLC, TX, USA). The normality of continuous variables was assessed using histograms and Q-Q plots. Outcomes variables were normally distributed. For consistency all continuous variables were described as mean and standard deviation. Binary outcomes were described as N (percentages) (**Table 1**). Comparisons between the intervention and control group and between the two ethnicity group categories (low/high-risk) were done using the unpaired t-test for normally distributed continuous variables, the Mann-Whitney test for continuous variables with non-normal distribution and the Fisher’s exact test for binary variables. In all analyses, predictors and outcomes did not differ in the groups (intervention *vs* control group, low *vs* high ethnicity group category) and the effect sizes were similar. Therefore, women from intervention and control groups and of low- and high-risk ethnicity were pooled together and adjusted for group allocation in all analyses. All analyses were also adjusted for infant age and sex, where appropriate.

**Table 1:** Maternal and offspring characteristics.

Maternal Characteristics	Mean ± SD	Infant characteristics	Mean ± SD
Number of patients	193	**Birth anthropometric parameters**	
Age (years)	33.6 ± 4.8	Number of patients (N, %)	190 (Male:52)
High risk ethnicity (yes; N(%))	39 (22.7)	Gestational age (weeks)	39.7 ± 1.1
Personal history of GDM (yes; N(%))	24 (21.4)	Weight (kg)	3.4 ± 0.46
Pre-pregnancy BMI (kg/m^2^)	25.9 ± 5.1	Length z-score (SD) ^1^	0.10 ± 1.4
Gestational weight gain (kg)	12.6 ± 6.5	Weight z-score (SD) ^1^	0.18 ± 1.1
Gestational age at the 1^st^ GDM visit (weeks)	29.0 ± 2.4	BMI (kg/m2)	13.7 ± 1.7
Weight at the 1^st^ GDM visit (kg)	79.6 ± 14.7	LGA ^1,2^	22 (11.8%)
Fat mass by BIA at the 1^st^ GDM visit (kg)	31.9 ± 9.5	SGA ^1,3^	20 (10.8%)
**Maternal metabolic parameters**			
Glucose at the 1^st^ GDM visit (mmol/L)	4.9 ± 0.49	**6-8 weeks anthropometric parameters**	
Insulin levels at the 1^st^ GDM visit (mIU/L)	16.1 ± 8.2	Number of patients (N, %)	185 (Male:51)
HOMA-IR at the 1^st^ GDM visit	3.6 ± 2.1	Age (days)	44.7 ± 9.3
QUICKI at the 1^st^ GDM visit	0.24 ± 0.03	Weight z-score (SD) ^4^	-0.12 ± 1.0
HbA1c at the 1^st^ GDM visit (%)	5.1 ± 0.31	Length z-score (SD) ^4^	0.06 ± 1.3
HbA1c at the 1^st^ GDM visit (mmol/mol)	32.2 ± 2.0	BMI (kg/m2)	15.2 ± 2.0
Triglycerides at the 1^st^ GDM visit (mmol/L)	2.4 ± 0.77	BMI z-score (SD) ^4^	-0.2 ± 1.3
HDL at the 1^st^ GDM visit (mmol/L)	1.8 ± 0.38	BMI z-score >1SD	20 (11.1%)
Gestational age at the last GDM visit (weeks)	36.9 ± 1.2	Sum of 4 skinfolds (mm)	34.5 ± 7.5
HbA1c at the last GDM visit (%)	5.3 ± 0.28		
HbA1c at the last visit (mmol/mol)	34.4 ± 1.8	**1 year anthropometric parameters**	
**Fetal parameters**		Number of patients (N, %)	170 (Male:52)
Number of patients	46	Age (months)	12.4 ± 1.0
Cord blood glucose (mmol/L)	4.0 ± 1.1	Weight z-score (SD) ^4^	0.32 ± 0.91
Cord blood insulin (mIU/L)	11.6 ± 9.4	Length z-score (SD) ^4^	0.27 ± 1.2
Cord blood C-Peptide (μg/L)	1.6 ± 0.76	BMI (kg/m2)	16.9 ± 1.6
Cord blood HOMA-IR	2.1 ± 0.16	BMI z-score (SD) ^4^	0.23 ± 1.1
Cord blood triglycerides (mmol/L)	0.53 ± 0.38	BMI z-score >1SD ^4^	39 (24.4%)
Cord blood HDL (mmol/L)	0.76 ± 0.22	Sum of 4 skinfolds (mm)	38.2 ± 10.6

GDM gestational diabetes mellitus, BMI body mass index, BIA Bioelectrical impedance analysis, HOMA-IR Homeostatic Model Assessment for Insulin Resistance, QUICKI quantitative insulin-sensitivity check index, HbA1c glycated hemoglobin, HDL high density lipoprotein, SD standard deviation, LGA large for gestational age, SGA small for gestational age.

^1^ according to the Intergrowth 21^st^ newborn size application tool (39).

^2^ LGA: birth weight >90th percentile for sex and gestational age using the Intergrowth 21^st^ newborn size application tool (39).

^3^ SGA: birth weight <10th percentile for sex and gestational age using the Intergrowth newborn size application tool (39).

^4^ according to the WHO Anthro Survey Analyser tool (40).

We initially performed univariate linear and logistic regression analyses with infant anthropometric parameters as the dependent variables (**Supplementary Tables 1–3**).

Maternal and fetal (cord blood) predictors with a p-value < 0.05 in univariate analysis were included in stepwise multiple regression analyses models. We performed three different multivariate models, a first one including both maternal and fetal predictors, and a second and third one including only maternal or only fetal predictors, respectively. Fetal predictors were available for N = 46 participants. These analyses were adjusted for group allocation, infant age and sex where appropriate, as well as maternal ethnicity group category, parity and maternal age. These analyses were performed in order to identify the most significant maternal and fetal predictors of infant anthropometric parameters at birth, 6-8 weeks and 1 year (**Table 2**). We tested for collinearity for all predictors and separate models were performed for predictors with a collinearity index ≥ 0.6. More specifically, as the collinearity index was ≥ 0.6 between HbA1c at the 1^st^ and last GDM visit, between pre-pregnancy BMI, weight and fat mass by BIA at the 1^st^ GDM visit, between insulin, HOMA-IR and QUICKI, as well as between cord blood insulin, C-peptide and HOMA-IR, separate multivariate models were performed for these predictors if more than one of them were significantly related to the respective outcome variable in univariate analyses (see **Supplementary Tables 1–3**). For all analyses, β-coefficients (for continuous outcomes) and adjusted odds ratios (aORs-for binary outcomes) are reported along with their 95% confidence intervals (CIs), and statistical significance was set at 0.05.

**Table 2:** Maternal and fetal predictors of infant anthropometry in multivariate regression analysis.

Offspring anthropometry	Predictors	OR ^4^/β-coefficient	95% CI		p-value
**Birth**					
	**Maternal and Fetal (BF)**				
Weight z-score (SD) ^1^	QUICKI at the 1^st^ GDM visit	-20.87	-38.40	-3.34	0.023
	Cord blood HDL (mmol/l)	3.77	1.33	6.21	0.005
	**Maternal**				
BMI (kg/m2)	HDL at the 1^st^ GDM visit (mmol/l)	-1.55	-2.52	-0.58	0.002
LGA^1,2^	HbA1c at the 1^st^ GDM visit (%) ^4^	5.45	1.04	28.66	0.045
	**Fetal**				
Weight z-score (SD) ^1^	Cord blood HDL (mmol/l)	1.61	0.25	0.30	0.022
**6-8 weeks**					
	**Maternal and Fetal (BF)**				
BMI (kg/m2)	Gestational weight gain (kg)	0.09	0.03	0.15	0.006
	**Maternal**				
BMI (kg/m2)	Gestational weight gain (kg)	0.06	0.01	0.12	0.023
Sum of 4 Skinfolds (mm)	HDL at the 1^st^ GDM visit (mmol/l)	-3.86	-7.17	-0.55	0.023
	**Fetal**				
BMI (kg/m2)	Cord blood insulin (mIU/L)	0.07	0.01	0.12	0.022
**1 year**					
	**Maternal and Fetal (BF)**				
BMI z-score (SD) ^3^	Cord blood HOMA-IR	-0.37	-0.07	-0.05	0.026
Sum of 4 Skinfolds (mm)	HbA1c at the 1^st^ GDM visit (%)	11.64	0.527	22.4	0.041
	Cord blood insulin (mIU/L)	-0.62	-1.04	-0.21	0.005
	Cord blood C-peptide (μg/L)	-8.65	-13.86	-3.45	0.002
	Cord blood HOMA-IR	-3.72	-6.45	-1.00	0.009
	**Maternal**				
Weight z-score (SD) ^3^	Weight at the 1^st^ GDM visit (kg)	0.01	0.00	0.02	0.017
	HbA1c at the 1^st^ GDM visit (%)	0.54	0.03	1.05	0.038
BMI (kg/m^2^)	Weight at the 1^st^ GDM visit (kg)	0.02	0.00	0.04	0.022
	HbA1c at the last GDM visit (%) ^5^	1.26	0.15	2.36	0.027
	HbA1c at the 1^st^ GDM visit (%) ^5^	0.93	0.03	0.82	0.043
BMI z-score (SD) ^3^	Pre-pregnancy BMI (kg/m^2^) ^5^	0.03	0.00	0.07	0.037
	Weight at the 1^st^ GDM visit (kg) ^5^	0.02	0.00	0.03	0.010
	Fat mass by BIA at the 1st GDM visit (kg) ^5^	0.02	0.00	0.04	0.033
	**Fetal**				
BMI z-score (SD) ^3^	Cord blood HOMA-IR	-0.37	-0.70	-0.05	0.026
Sum of 4 Skinfolds (mm)	Cord blood insulin (mIU/L) ^5^	-0.68	-1.11	-0.25	0.003
	Cord blood C-peptide (μg/L) ^5^	-8.65	-13.86	-3.45	0.002
	Cord blood HOMA-IR ^5^	-3.72	-6.45	-1.00	0.009

OR Odds Ratio, CI Confidence Interval, BMI body mass index, GDM gestational diabetes mellitus, BIA Bioelectrical impedance analysis, HOMA-IR Homeostatic Model Assessment for Insulin Resistance, QUICKI quantitative insulin-sensitivity check index, HbA1c glycated hemoglobin, HDL high density lipoprotein, SD standard deviation, LGA large for gestational age, SGA small for gestational age.

^1^ according to the Intergrowth 21^st^ newborn size application tool (39).

^2^ LGA: birth weight >90th percentile for sex and gestational age using the Intergrowth 21st newborn size application tool (39).

^3^ according to the WHO Anthro Survey Analyser tool (40).

^4^ this value corresponds to an OR.

^5^ separate models were performed due to high collinearity (collinearity index ≥ 0.6) between predictors.

Stepwise multiple logistic regression analyses, adjusted for maternal age, ethnicity (high/low risk), parity, allocation group (intervention/control), infant sex and age (where appropriate). Outcomes are only shown if at least one predictor is found. Only significant results are displayed (defined significance, p-value <0.05, see text). Three distinct models were performed (combined model, including maternal and fetal predictors, model including only maternal or only fetal predictors), and results are displayed separately.

## 3 Results

The initial population included 211 women with GDM participating in the randomized-controlled intervention, and their offspring. One woman was excluded because the diagnosis of GDM was done before 13 weeks of gestation, and 17 were excluded due to multiple gestation (N=4), and/or because their offspring were born premature (< 37 weeks of gestational age, N=16). Thus, 193 women (93 intervention group/100 control group) were included in the final analysis.

### 3.1 Maternal, fetal and infant characteristics

Detailed information on the maternal characteristics, cord blood metabolic parameters, and offspring anthropometric outcomes at birth, 6-8 weeks, and 1 year are shown in **Table 1**. Briefly, mean maternal age was 33.6 ± 4.8 years, pre-pregnancy BMI was 25.9 ± 5.1 kg/m^2^ and GWG 12.6 ± 6.5 kg. GA at birth was 39.7 ± 1.1 weeks, mean weight z-score at birth was +0.18 ± 1.1 standard deviations (SD) and 11.8% of newborns were LGA. At 6-8 weeks and 1 year, mean offspring BMI z-score was -0.2 ± 1.3 and +0.23 ± 1.1 SD respectively.

### 3.2 Associations between maternal and fetal predictors and offspring anthropometry at birth, 6-8 weeks, and 1 year in univariate analyses

Different maternal metabolic and fetal predictors were associated with offspring outcomes at birth, while maternal anthropometric and metabolic as well as fetal predictors were associated with offspring outcomes at 6-8 weeks and 1 year in univariate analyses (**Supplementary Tables 1–3**).

### 3.3 Associations between maternal and fetal predictors and offspring anthropometry at birth, 6-8 weeks and 1 year in multivariate analyses

The significant results of all multivariate univariate analyses are shown in **Table 2**.

#### 3.3.1 Birth

In the models including only maternal predictors, HDL at the 1^st^ GDM visit was negatively associated with offspring BMI, and HbA1c at the 1^st^ GDM visit was positively associated with LGA (p ≤0.045). In the models including only fetal predictors, cord blood HDL was positively associated with the weight z-score (p ≤0.022). In the combined maternal and fetal model, maternal QUICKI at the 1^st^ GDM visit was negatively associated and cord blood HDL was positively associated with the weight z-score (p ≤0.023).

#### 3.3.2 6-8 weeks

In the models including only maternal predictors, HDL at the 1^st^ GDM visit was negatively associated with the sum of skinfolds, whereas GWG showed a positive association with BMI (both p =0.023). In the model including only fetal predictors, cord blood insulin presented a positive association with offspring BMI (p =0.022). Lastly, in the combined model, GWG was positively associated with BMI (p =0.006).

#### 3.3.3 1 year

In the models including only maternal predictors, pre-pregnancy BMI, as well as weight and fat mass at the 1^st^ GDM visit showed a positive association with the offspring BMI z-score (all p ≤0.037). Weight at the 1^st^ GDM visit and HbA1c at the 1^st^ and last GDM visit presented a positive association with BMI (both p ≤0.043). Moreover, weight and HbA1c at the 1^st^ GDM visit were positively correlated the weight z-score (both p ≤0.038). In models including only fetal predictors, cord blood HOMA-IR, C-peptide and insulin showed a negative association with the sum of 4 skinfolds (all p ≤0.009). Cord blood HOMA-IR presented a negative association with the BMI z-score (p =0.026). In the combined maternal and fetal models, HbA1c at the 1^st^ GDM visit showed a positive, whereas cord blood insulin, C-peptide and HOMA-IR a negative association with the sum of 4 skinfolds (all p ≤0.041). Finally, cord blood HOMA-IR was negatively associated with the BMI z-score (p =0.026).

## 4 Discussion

This prospective, observational study of women with GDM and their offspring found that maternal anthropometric, metabolic and fetal metabolic parameters distinctively predicted offspring anthropometry during the 1^st^ year of life. Maternal metabolic parameters during the 3^rd^ trimester including QUICKI and HDL were negatively associated with offspring anthropometry at birth;HDL was negatively associated with offspring anthropometry at 6-8 weeks,whereas HbA1c was positively associated with offspring anthropometry at 1 year. Maternal anthropometric parameters, such as pre-pregnancy BMI, weight and fat mass during the 3^rd^ trimester, and GWG predicted higher offspring anthropometry, but only after birth. Cord blood HDL correlated positively with weight z-score at birth, while cord blood insulin, C-peptide and HOMA-IR were associated with anthropometry during the 1^st^ year of life in an age dependent pattern, showing positive associations at birth and 6-8 weeks and negative associations at 1 year.

### 4.1 Impact of maternal and fetal metabolism on birth anthropometry

Maternal HDL and QUICKI at the 1^st^ GDM visit, both observed in situations of increased insulin sensitivity, were negatively associated with weight z-score and BMI respectively. In contrast, no associations were found between maternal HDL levels and birth anthropometry in another population with GDM (19). However, they found that maternal triglyceride levels were positively associated with adjusted birth weight centiles and LGA in their insulin-treated subpopulation; similar results have been documented in studies in the general pregnant population (19, 22, 23). In insulin-resistant states, when triglycerides are elevated, HDL is often decreased and may be a more stable marker than triglycerides (43). An adverse maternal lipid profile programs offspring regarding obesity in human and animal studies at and beyond birth, by multiple mechanisms including the offspring’s eating behavior and energy expenditure, adipocyte development, genetics, epigenetics and shared post-natal environment and the inverse could be true for markers of favorable lipid profiles such as higher HDL (44, 45). HbA1c reflects overall maternal and thus subsequent fetal glucose exposure and was positively associated with LGA. This is in agreement with previous data in women with GDM (19), but has not been found in all studies (20, 21). As increased adiposity at birth represents a risk factor for obesity and the metabolic syndrome later in life (46), higher HbA1c levels, but also lower HDL levels in pregnancy may be used for risk stratification in these pregnancies with the aim to reduce the metabolic risk of the offspring (21). In contrast to previous studies in GDM populations, maternal anthropometric parameters had no impact on birth anthropometry (19–21). This may be explained by the smaller variability of maternal anthropometric parameters in our cohort and the fact that the impact of maternal anthropometry may be reduced by the strict monitoring, which is represented by the low % of LGA in our sample.

In terms of fetal metabolism, cord blood HDL showed a positive association with birth weight z-score, in agreement with a study in the general population (25), but on contrast to other studies (19, 23). Interestingly, cord blood HDL influenced birth anthropometry independently from maternal parameters in our study, highlighting the importance of fetal lipid metabolism for fetal growth, that might be distinct from, albeit also dependent of, the impact of maternal parameters. The findings of two previous studies in GDM showing inverse correlations between cord blood triglyceride and birth anthropometry further underline our results (19, 28). Large molecules such as HDL cannot cross the placenta directly, but they could affect the metabolism of other lipids, depending on the concentration and presence (47, 48). As proposed by Ye et al. (48), this potential interaction between lipid particles in the fetus could be critical for the fetal and neonatal metabolism, and may have a lasting impact on the metabolic health of the offspring. Cord blood markers including insulin, C-peptide and HOMA-IR were related to birth anthropometry in univariate, but not in multivariate analyses. Studies in healthy pregnancies, with mild untreated hyperglycemia (HAPO) or GDM have found a positive association between cord blood insulin and/or C-peptide and birth anthropometry, but they did not adjust for cord blood HDL (19, 24, 25, 27, 28). In agreement with previous studies, cord blood glucose was not related to birth anthropometry (26, 28).

### 4.2 Impact of maternal and fetal metabolism on infant anthropometry at 6-8 weeks of life

GWG was positively associated with infant BMI and maternal HDL negatively associated with the sum of skinfolds at 6-8 weeks in multivariate analyses (maternal and combined model). Maternal metabolic parameters related to insulin resistance were only correlated to higher anthropometric parameters in univariate analyses. Similarly, another study also found no relationship between maternal 3^rd^ trimester C-peptide levels and the offspring’s adipose tissue at 6 weeks and 4 months of age in adjusted models (49). Lastly, our study found a positive association between cord blood insulin and infant BMI.

### 4.3 Impact of maternal and fetal metabolism on infant anthropometry at 1 year

Regarding maternal metabolic parameters, HbA1c at the 1^st^ GDM visit was positively associated with weight z-score, BMI, and the sum of skinfolds and HbA1c at the last GDM visit was positively associated with BMI at 1 year. Similarly, the HAPO-FUS study found a positive association between 3^rd^ trimester HbA1c and offspring anthropometry, including BMI, body fat and the presence of overweight/obesity later in childhood, at 10-14 years (16).

Maternal anthropometric parameters, including pre-pregnancy BMI, 1^st^ GDM visit weight and fat mass were also positively associated with weight z-score, BMI and BMI z-score at 1 year. This is in accordance with data in the general population where higher pre-pregnancy BMI was associated with higher offspring BMI and adiposity at 4-7 years (16, 44, 50). These findings may be explained by fetal programming and/or lifestyle and genetic characteristics (50, 51).

In terms of fetal metabolism, cord blood insulin, C-Peptide, and HOMA-IR were inversely associated with infant BMI z-score and the sum of skinfolds at 1 year, in agreement with a study in the general population which found a negative association between cord blood insulin and the sum of 4 skinfolds and % body fat at 3 years of age, but not in older children (52). Similarly, another study showed an inverse association between cord blood C-peptide and weight at 1 year in girls (53). In contrast, other studies in the general population have found a positive or no correlation between cord blood insulin and infant anthropometry or adiposity at 1-2 years (54, 55). Lastly, cord blood C-peptide was positively associated with offspring adiposity parameters at a mean age of 11.4 years in the HAPO Study and FUS (56).

The switch in the effect of cord blood insulin, C-peptide and HOMA-IR on offspring anthropometry during the 1^st^ years of life (positive association at birth and 1^st^ months of life, negative association at 1 year and the positive association in later childhood and adolescence) is intriguing and needs more research. A hypothesis for our findings is that at birth and the 1^st^ months of life cord blood insulin, C-peptide and HOMA-IR may be markers of metabolic (glucose) overload in these babies and thus the impact of the maternal metabolism and fuel overload is significant. This may lead to fat accretion and body fat accumulation (mostly maternally-driven and not a clear marker of initial fetal or infant insulin resistance). However, as infants of mothers with GDM are insulin resistant and get even more insulin-resistant with increasing fat accretion, the insulin resistance at the adipose tissue level, could then prevent further fat accumulation in the subcutaneous adipose tissue, and might foster fat deposition in ectopic tissues (57). Thereby, fat cell lipolysis may also play a role (57). Further investigation is necessary to unravel these pathophysiological mechanisms. In later childhood, mechanisms related to excess energy intake and insulin resistance could again become more dominant for fat accretion.

### 4.4 Strengths and limitations

This is one of the rare studies assessing the impact of maternal and fetal parameters on infant anthropometric and adiposity parameters at different time points during the 1^st^ year of life in pregnancies with GDM. Its prospective nature allowed us to include detailed information on various maternal and fetal parameters and to assess complex associations between maternal and fetal metabolism and growth during the 1^st^ year of life. However, some limitations can also be noted. Cord blood parameters were available for a small proportion of our population (46 patients), which may have under or overestimated some of our correlations. Moreover, due to the small sample size, particularly regarding cord blood parameters, we were not able to perform separate analyses for the intervention and control group or according to low and high-risk ethnicity. However, maternal and fetal predictors and infant anthropometric parameters were not different between groups. Moreover, we did not assess the correlation between fetal anthropometry and birth and infancy anthropometry as the majority of fetal ultrasounds were not performed at our center. Lastly, collinearity was present between multiple maternal and fetal predictors. Therefore, in multivariate analyses, multiple testing models were necessary, which may have under- or overestimated some of our results.

## 5 Conclusions

Maternal anthropometric, metabolic and fetal metabolic parameters distinctively influenced offspring anthropometry during the 1^st^ year of life and this in an age-dependent manner. Distinct age-dependent associations were particularly present for cord blood metabolic parameters. These observations show the complexity of the pathophysiological mechanism for the developing offspring and need further investigation. In the future, these predictors could be used for a more personalized follow-up of women with GDM and their offspring, to reduce the risks associated with an unfavorable *in-utero* environment and to foster a favorable fetal programming.

## Data availability statement

The original contributions presented in the study are included in the article/**Supplementary materials**. Further inquiries can be directed to the corresponding author.

## Ethics statement

The studies involving human participants were reviewed and approved by the Human Research Ethics Committee of the Canton de Vaud (study number 2016-00745). The study was conducted in accordance with the guidelines of the declaration of Helsinki, and good clinical practice. Written informed consent to participate in this study was provided by the participants’ legal guardian/next of kin.

## Author contributions

JP and AH conceived the study, designed the trial, obtained grant funding, and oversaw management of the trial. LG and DQ helped in designing parts of the study and participated in the implementation of the study. M-CA performed the data analysis, and interpretation and wrote the draft manuscript, under the supervision of JP. AL and BS participated in data analysis. DQ, LG, AH and JP revised the manuscript for important intellectual content and gave final approval for the version to be published. All authors contributed to the article and approved the submitted version.
