# Supplementary material for: Maternal and fetal predictors of anthropometry in the first year of life in offspring of women with GDM
Source: Front Endocrinol (Lausanne). 2023 Mar 28;14:1144195. doi: 10.3389/fendo.2023.1144195 (PMC10086315; doi:10.3389/fendo.2023.1144195)
Supplement: Supplementary file 1 [file Table_1.docx]

Supplementary Material

Maternal and fetal predictors of anthropometry in the first year of life in offspring of women with GDM

**Maria-Christina Antoniou ^1^ #, Dan Yedu Quansah ^2^ #, Suzanne Mühlberg ^3^, Leah Gilbert ^2^ Amar Arhab ^2^, Sybille Schenk ^2^, Alain Lacroix ^2^, Bobby Stuijfzand ^2^, Antje Horsch ^4,5^ *, Jardena Jacqueline Puder ^2^ ***

^1^ Unit of Pediatric Endocrinology and Diabetology, Pediatric Service, Woman-Mother-Child Department, Lausanne University Hospital, Avenue Pierre-Decker 2, 1011 Lausanne.

^2^ Obstetric Service, Woman-Mother-Child Department, Lausanne University Hospital, Avenue Pierre-Decker 2, 1011 Lausanne, Switzerland.

^3^ Faculty of Biology and Medicine, University of Lausanne, 1011 Lausanne, Switzerland.

^4^ Institute of Higher Education and Research in Healthcare (IUFRS), University of Lausanne, Route de la Corniche 10, 1010 Lausanne, Switzerland

^5^ Neonatology Service, Woman-Mother-Child Department, Lausanne University Hospital, Avenue Pierre-Decker 2, 1011 Lausanne, Switzerland.

#These authors contributed equally to this work and share first authorship

* These authors contributed equally to this work and share last authorship

***Correspondence:**

*****Corresponding author: Prof. Jardena Puder; e-mail: [jardena.puder@chuv.ch](mailto:jardena.puder@chuv.ch)

Supplementary table 1. Maternal and fetal predictors of anthropometry at birth in univariate regression analysis

| **Birth Anthropometric Parameters** |  | **Predictors** | **OR ^4^/** | **95% CI** | | **p-value** |
| --- | --- | --- | --- | --- | --- | --- |
|  |  |  | **β-Coefficient** |  |  |  |
| Weight (kg) | Maternal Predictors | Prepregnancy BMI (kg/m^2^) | 0.004 | -0.008 | 0.015 | 0.519 |
|  |  | Gestational weight gain (kg) | 0.009 | -0.002 | 0.020 | 0.094 |
|  |  | Weight at the 1^st^ GDM visit (kg) | 0.003 | -0.001 | 0.007 | 0.173 |
|  |  | Fat mass by BIA at the 1^st^ GDM visit (kg) | 0.004 | -0.003 | 0.010 | 0.297 |
|  |  | Glucose at the 1^st^ GDM visit (mmol/L) | 0.077 | -0.053 | 0.207 | 0.243 |
|  |  | Insulin at the 1^st^ GDM visit (mIU/L) | 0.007 | -0.002 | 0.015 | 0.121 |
|  |  | HOMA-IR at the 1^st^ GDM visit | 0.027 | -0.007 | 0.060 | 0.118 |
|  |  | QUICKI at the 1^st^ GDM visit | -1.920 | -4.040 | 0.201 | 0.076 |
|  |  | HbA1c at the 1^st^ GDM visit (%) | 0.224 | 0.014 | 0.434 | 0.037 |
|  |  | HbA1c at the last GDM visit (%) | 0.388 | 0.146 | 0.629 | 0.002 |
|  |  | Triglycerides at the 1^st^ GDM visit (mmol/L) | 0.022 | -0.063 | 0.110 | 0.612 |
|  |  | HDL at the 1^st^ GDM visit (mmol/L) | -0.126 | -0.300 | 0.047 | 0.153 |
|  | Fetal Predictors | Cord blood glucose (mmol/L) | -0.058 | -0.188 | 0.072 | 0.372 |
|  |  | Cord blood insulin (mIU/L) | 0.020 | 0.006 | 0.035 | 0.006 |
|  |  | Cord blood C-Peptide (μg/L) | 0.234 | 0.047 | 0.421 | 0.016 |
|  |  | Cord blood HOMA-IR | 0.088 | 0.019 | 0.156 | 0.014 |
|  |  | Cord blood triglycerides (mmol/L) | -0.305 | -0.727 | 0.117 | 0.152 |
|  |  | Cord blood HDL (mmol/L) | 0.664 | 0.030 | 1.299 | 0.041 |
| Weight z-score (SD) ^1^ | Maternal Predictors | Prepregnancy BMI (kg/m^2^) | 0.008 | -0.019 | 0.035 | 0.557 |
|  |  | Gestational weight gain (kg) | 0.022 | -0.004 | 0.047 | 0.092 |
|  |  | Weight at the 1^st^ GDM visit (kg) | 0.008 | -0.003 | 0.018 | 0.138 |
|  |  | Fat mass by BIA at the 1^st^ GDM visit (kg) | 0.010 | -0.006 | 0.026 | 0.234 |
|  |  | Glucose at the 1^st^ GDM visit (mmol/L) | 0.193 | -0.120 | 0.506 | 0.226 |
|  |  | Insulin at the 1^st^ GDM visit (mIU/L) | 0.020 | -0.001 | 0.040 | 0.057 |
|  |  | HOMA-IR at the 1^st^ GDM visit | 0.077 | -0.003 | 0.157 | 0.058 |
|  |  | QUICKI at the 1^st^ GDM visit | -5.331 | -10.425 | -0.237 | 0.040 |
|  |  | HbA1c at the 1^st^ GDM visit (%) | 0.585 | 0.094 | 1.076 | 0.020 |
|  |  | HbA1c at the last GDM visit (%) | 0.928 | 0.351 | 1.505 | 0.002 |
|  |  | Triglycerides at the 1^st^ GDM visit (mmol/L) | 0.078 | -0.126 | 0.281 | 0.453 |
|  |  | HDL at the 1^st^ GDM visit (mmol/L) | -0.352 | -0.766 | 0.063 | 0.096 |
|  | Fetal Predictors | Cord blood glucose (mmol/L) | -0.146 | -0.449 | 0.157 | 0.336 |
|  |  | Cord blood insulin (mIU/L) | 0.043 | 0.011 | 0.075 | 0.010 |
|  |  | Cord blood C-Peptide (μg/L) | 0.519 | 0.079 | 0.958 | 0.022 |
|  |  | Cord blood HOMA-IR | 0.189 | 0.032 | 0.346 | 0.020 |
|  |  | Cord blood triglycerides (mmol/L) | -0.616 | -1.471 | 0.240 | 0.154 |
|  |  | Cord blood HDL (mmol/L) | 1.610 | 0.190 | 3.029 | 0.027 |
| BMI (kg/m^2^) | Maternal Predictors | Prepregnancy BMI (kg/m^2^) | 0.037 | -0.007 | 0.081 | 0.098 |
|  |  | Gestational weight gain (kg) | 0.023 | -0.020 | 0.067 | 0.292 |
|  |  | Weight at the 1^st^ GDM visit (kg) | 0.015 | -0.002 | 0.031 | 0.083 |
|  |  | Fat mass by BIA at the 1^st^ GDM visit (kg) | 0.022 | -0.004 | 0.047 | 0.102 |
|  |  | Glucose at the 1^st^ GDM visit (mmol/L) | 0.395 | -0.111 | 0.901 | 0.125 |
|  |  | Insulin at the 1^st^ GDM visit (mIU/L) | 0.049 | 0.016 | 0.081 | 0.004 |
|  |  | HOMA-IR at the 1^st^ GDM visit | 0.197 | 0.070 | 0.324 | 0.003 |
|  |  | QUICKI at the 1^st^ GDM visit | -8.663 | -16.840 | -0.487 | 0.038 |
|  |  | HbA1c at the 1^st^ GDM visit (%) | 0.961 | 0.142 | 1.779 | 0.022 |
|  |  | HbA1c at the last GDM visit (%) | 1.247 | 0.206 | 2.288 | 0.019 |
|  |  | Triglycerides at the 1^st^ GDM visit (mmol/L) | 0.032 | -0.300 | 0.364 | 0.849 |
|  |  | HDL at the 1^st^ GDM visit (mmol/L) | -0.708 | -1.382 | -0.034 | 0.040 |
|  | Fetal Predictors | Cord blood glucose (mmol/L) | -0.375 | -0.772 | 0.022 | 0.063 |
|  |  | Cord blood insulin (mIU/L) | 0.053 | 0.008 | 0.098 | 0.023 |
|  |  | Cord blood C-Peptide (μg/L) | 0.543 | -0.060 | 1.145 | 0.076 |
|  |  | Cord blood HOMA-IR | 0.197 | -0.024 | 0.419 | 0.079 |
|  |  | Cord blood triglycerides (mmol/L) | -1.232 | -2.513 | 0.049 | 0.059 |
|  |  | Cord blood HDL (mmol/L) | 1.072 | -0.969 | 3.114 | 0.295 |

| LGA ^1,2^ | Maternal Predictors | Prepregnancy BMI (kg/m^2^) ^4^ | -0.027 | -0.111 | 0.057 | 0.530 |
| --- | --- | --- | --- | --- | --- | --- |
|  |  | Gestational weight gain (kg) ^4^ | 0.037 | -0.038 | 0.112 | 0.330 |
|  |  | Weight at the 1^st^ GDM visit (kg) ^4^ | 0.001 | -0.028 | 0.031 | 0.931 |
|  |  | Fat mass by BIA at the 1^st^ GDM visit (kg) ^4^ | -0.005 | -0.052 | 0.042 | 0.844 |
|  |  | Glucose at the 1^st^ GDM visit (mmol/L) ^4^ | 0.405 | -0.443 | 1.253 | 0.349 |
|  |  | Insulin at the 1^st^ GDM visit (mIU/L) ^4^ | 0.031 | -0.020 | 0.082 | 0.231 |
|  |  | HOMA-IR at the 1^st^ GDM visit ^4^ | 0.134 | -0.059 | 0.326 | 0.174 |
|  |  | QUICKI at the 1^st^ GDM visit ^4^ | -7.735 | -22.700 | 7.231 | 0.311 |
|  |  | HbA1c at the 1^st^ GDM visit (%) ^4^ | 1.761 | 0.284 | 3.239 | 0.019 |
|  |  | HbA1c at the last GDM visit (%) ^4^ | 2.597 | 0.565 | 4.630 | 0.012 |
|  |  | Triglycerides at the 1^st^ GDM visit (mmol/L) ^4^ | 0.207 | -0.339 | 0.752 | 0.458 |
|  |  | HDL at the 1^st^ GDM visit (mmol/L) ^4^ | -0.640 | -1.879 | 0.598 | 0.311 |
|  | Fetal Predictors | Cord blood glucose (mmol/L) ^4^ | 0.227 | -0.737 | 1.190 | 0.645 |
|  |  | Cord blood insulin (mIU/L) ^4^ | 0.069 | -0.017 | 0.155 | 0.114 |
|  |  | Cord blood C-Peptide (μg/L) ^4^ | 1.089 | -0.159 | 2.338 | 0.087 |
|  |  | Cord blood HOMA-IR ^4^ | 0.293 | -0.100 | 0.686 | 0.144 |
|  |  | Cord blood triglycerides (mmol/L) ^4^ | 0.604 | -1.577 | 2.784 | 0.587 |
|  |  | Cord blood HDL (mmol/L) ^4^ | 2.428 | -2.076 | 6.931 | 0.291 |
| SGA ^1,3^ | Maternal Predictors | Prepregnancy BMI (kg/m^2^) ^4^ | -0.012 | -0.097 | 0.073 | 0.777 |
|  |  | Gestational weight gain (kg) ^4^ | -0.013 | -0.094 | 0.068 | 0.746 |
|  |  | Weight at the 1^st^ GDM visit (kg) ^4^ | -0.014 | -0.047 | 0.019 | 0.411 |
|  |  | Fat mass by BIA at the 1^st^ GDM visit (kg) ^4^ | -0.023 | -0.074 | 0.029 | 0.389 |
|  |  | Glucose at the 1^st^ GDM visit (mmol/L) ^4^ | -0.962 | -2.118 | 0.193 | 0.103 |
|  |  | Insulin at the 1^st^ GDM visit (mIU/L) ^4^ | -0.019 | -0.085 | 0.046 | 0.561 |
|  |  | HOMA-IR at the 1^st^ GDM visit ^4^ | -0.121 | -0.403 | 0.160 | 0.397 |
|  |  | QUICKI at the 1^st^ GDM visit ^4^ | 8.907 | -5.662 | 23.476 | 0.231 |
|  |  | HbA1c at the 1^st^ GDM visit (%) ^4^ | 0.035 | -1.456 | 1.526 | 0.963 |
|  |  | HbA1c at the last GDM visit (%) ^4^ | -0.086 | -1.964 | 1.792 | 0.928 |
|  |  | Triglycerides at the 1^st^ GDM visit (mmol/L) ^4^ | 0.169 | -0.407 | 0.744 | 0.566 |
|  |  | HDL at the 1^st^ GDM visit (mmol/L) ^4^ | 0.829 | -0.395 | 2.054 | 0.184 |
|  | Fetal Predictors | Cord blood glucose (mmol/L) ^4^ | -0.366 | -1.338 | 0.605 | 0.460 |
|  |  | Cord blood insulin (mIU/L) ^4^ | -0.063 | -0.214 | 0.087 | 0.411 |
|  |  | Cord blood C-Peptide (μg/L) ^4^ | -0.574 | -1.925 | 0.778 | 0.405 |
|  |  | Cord blood HOMA-IR ^4^ | -0.300 | -1.088 | 0.488 | 0.456 |
|  |  | Cord blood triglycerides (mmol/L) ^4^ | 1.072 | -1.002 | 3.146 | 0.311 |
|  |  | Cord blood HDL (mmol/L) ^4^ | -3.479 | -8.597 | 1.640 | 0.183 |

Abbreviations: *OR* Odds Ratio, *CI* Confidence Interval, *BMI* body mass index, *GDM* gestational diabetes mellitus, *BIA* Bioelectrical impedance analysis, *HOMA-IR* Homeostatic Model Assessment for Insulin Resistance, *QUICKI* quantitative insulin-sensitivity check index, *HbA1c* glycated hemoglobin, *HDL* high density lipoprotein, *SD* standard deviation, LGA large for gestational age, SGA small for gestational age.

^1^ according to the Intergrowth 21^st^ newborn size application tool (41)

^2^ LGA: birth weight >90th percentile for sex and gestational age using the Intergrowth 21^st^ newborn size application tool (41)

^3^ SGA: birth weight ˂10th percentile for sex and gestational age using the Intergrowth 21^st^ newborn size application tool (41)

^4^ this value corresponds to an OR

Univariate linear and logistic regression analyses at birth, adjusted for group allocation (intervention/control) and infant sex and age (where appropriate).

Supplementary table 2. Maternal and fetal predictors of anthropometry at 2 months in univariate regression analysis

| **2 month anthropometric parameters** |  | **Predictors** | **β -Coefficient** | **95% CI** | | **p- value** | |
| --- | --- | --- | --- | --- | --- | --- | --- |
|  |  |  |  |  |  |  |  |
| Weight z-score (SD) ^1^ | Maternal Predictors | Prepregnancy BMI (kg/m^2^) | 0.028 | 0.002 | 0.055 | | 0.037 |
|  |  | Gestational weight gain (kg) | 0.025 | -0.001 | 0.051 | | 0.055 |
|  |  | Weight at the 1^st^ GDM visit (kg) | 0.017 | 0.007 | 0.027 | | 0.001 |
|  |  | Fat mass by BIA at the 1^st^ GDM visit (kg) | 0.025 | 0.010 | 0.041 | | 0.001 |
|  |  | Glucose at the 1^st^ GDM visit (mmol/L) | 0.274 | -0.033 | 0.581 | | 0.080 |
|  |  | Insulin at the 1^st^ GDM visit (mIU/L) | 0.021 | 0.002 | 0.040 | | 0.027 |
|  |  | HOMA-IR at the 1^st^ GDM visit | 0.080 | 0.007 | 0.154 | | 0.033 |
|  |  | QUICKI at the 1^st^ GDM visit | -5.926 | -10.611 | -1.240 | | 0.014 |
|  |  | HbA1c at the 1^st^ GDM visit (%) | 0.370 | -0.134 | 0.874 | | 0.149 |
|  |  | HbA1c at the last GDM visit (%) | 0.631 | -0.004 | 1.266 | | 0.051 |
|  |  | Triglycerides at the 1^st^ GDM visit (mmol/L) | -0.001 | -0.200 | 0.197 | | 0.989 |
|  |  | HDL at the 1^st^ GDM visit (mmol/L) | -0.343 | -0.747 | 0.061 | | 0.095 |
|  | Fetal Predictors | Cord blood glucose (mmol/L) | -0.111 | -0.357 | 0.134 | | 0.366 |
|  |  | Cord blood insulin (mIU/L) | 0.026 | -0.002 | 0.054 | | 0.067 |
|  |  | Cord blood C-Peptide (μg/L) | 0.324 | -0.064 | 0.711 | | 0.099 |
|  |  | Cord blood HOMA-IR | 0.111 | -0.023 | 0.246 | | 0.102 |
|  |  | Cord blood triglycerides (mmol/L) | 0.069 | -0.670 | 0.808 | | 0.851 |
|  |  | Cord blood HDL (mmol/L) | 0.388 | -0.872 | 1.647 | | 0.538 |
| BMI (kg/m^2^) | Maternal Predictors | Prepregnancy BMI (kg/m^2^) | 0.030 | -0.022 | 0.082 | | 0.253 |
|  |  | Gestational weight gain (kg) | 0.060 | 0.009 | 0.110 | | 0.021 |
|  |  | Weight at the 1^st^ GDM visit (kg) | 0.017 | -0.002 | 0.037 | | 0.079 |
|  |  | Fat mass by BIA at the 1^st^ GDM visit (kg) | 0.024 | -0.006 | 0.054 | | 0.115 |
|  |  | Glucose at the 1^st^ GDM visit (mmol/L) | 0.285 | -0.307 | 0.876 | | 0.344 |
|  |  | Insulin at the 1^st^ GDM visit (mIU/L) | 0.029 | -0.009 | 0.067 | | 0.134 |
|  |  | HOMA-IR at the 1^st^ GDM visit | 0.114 | -0.036 | 0.264 | | 0.136 |
|  |  | QUICKI at the 1^st^ GDM visit | -7.794 | -17.357 | 1.769 | | 0.109 |
|  |  | HbA1c at the 1^st^ GDM visit (%) | 0.573 | -0.406 | 1.551 | | 0.250 |
|  |  | HbA1c at the last GDM visit (%) | 1.055 | -0.193 | 2.303 | | 0.097 |
|  |  | Triglycerides at the 1^st^ GDM visit (mmol/L) | 0.017 | -0.364 | 0.398 | | 0.931 |
|  |  | HDL at the 1^st^ GDM visit (mmol/L) | -0.154 | -0.931 | 0.624 | | 0.697 |
|  | Fetal Predictors | Cord blood glucose (mmol/L) | -0.331 | -0.689 | 0.026 | | 0.068 |
|  |  | Cord blood insulin (mIU/L) | 0.058 | 0.016 | 0.099 | | 0.007 |
|  |  | Cord blood C-Peptide (μg/L) | 0.714 | 0.133 | 1.296 | | 0.017 |
|  |  | Cord blood HOMA-IR | 0.193 | -0.017 | 0.403 | | 0.070 |
|  |  | Cord blood triglycerides (mmol/L) | -0.929 | -2.146 | 0.289 | | 0.131 |
|  |  | Cord blood HDL (mmol/L) | -0.287 | -2.276 | 1.703 | | 0.772 |
| BMI z-score (SD) ^1^ | Maternal Predictors | Prepregnancy BMI (kg/m^2^) | 0.023 | -0.012 | 0.058 | | 0.205 |
|  |  | Gestational weight gain (kg) | 0.034 | 0.000 | 0.068 | | 0.049 |
|  |  | Weight at the 1^st^ GDM visit (kg) | 0.013 | -0.001 | 0.026 | | 0.060 |
|  |  | Fat mass by BIA at the 1^st^ GDM visit (kg) | 0.017 | -0.003 | 0.038 | | 0.095 |
|  |  | Glucose at the 1^st^ GDM visit (mmol/L) | 0.213 | -0.190 | 0.616 | | 0.298 |
|  |  | Insulin at the 1^st^ GDM visit (mIU/L) | 0.017 | -0.008 | 0.043 | | 0.179 |
|  |  | HOMA-IR at the 1^st^ GDM visit | 0.071 | -0.029 | 0.171 | | 0.165 |
|  |  | QUICKI at the 1^st^ GDM visit | -4.771 | -11.157 | 1.615 | | 0.142 |
|  |  | HbA1c at the 1^st^ GDM visit (%) | 0.445 | -0.213 | 1.103 | | 0.183 |
|  |  | HbA1c at the last GDM visit (%) | 0.711 | -0.125 | 1.547 | | 0.095 |
|  |  | Triglycerides at the 1^st^ GDM visit (mmol/L) | -0.025 | -0.283 | 0.234 | | 0.851 |
|  |  | HDL at the 1^st^ GDM visit (mmol/L) | -0.143 | -0.675 | 0.389 | | 0.596 |
|  | Fetal Predictors | Cord blood glucose (mmol/L) | -0.260 | -0.520 | 0.001 | | 0.051 |
|  |  | Cord blood insulin (mIU/L) | 0.035 | 0.005 | 0.064 | | 0.024 |
|  |  | Cord blood C-Peptide (μg/L) | 0.417 | -0.001 | 0.835 | | 0.051 |
|  |  | Cord blood HOMA-IR | 0.113 | -0.037 | 0.264 | | 0.135 |
|  |  | Cord blood triglycerides (mmol/L) | -0.304 | -1.108 | 0.500 | | 0.449 |
|  |  | Cord blood HDL (mmol/L) | -0.393 | -1.774 | 0.988 | | 0.568 |
| Sum of 4 Skinfolds (mm) | Maternal Predictors | Prepregnancy BMI (kg/m^2^) | 0.179 | -0.014 | 0.373 | | 0.069 |
|  |  | Gestational weight gain (kg) | 0.114 | -0.067 | 0.295 | | 0.215 |
|  |  | Weight at the 1^st^ GDM visit (kg) | 0.088 | 0.015 | 0.161 | | 0.019 |
|  |  | Fat mass by BIA at the 1^st^ GDM visit (kg) | 0.099 | -0.015 | 0.213 | | 0.089 |
|  |  | Glucose at the 1^st^ GDM visit (mmol/L) | 1.978 | -0.252 | 4.207 | | 0.082 |
|  |  | Insulin at the 1^st^ GDM visit (mIU/L) | 0.148 | 0.016 | 0.279 | | 0.028 |
|  |  | HOMA-IR at the 1^st^ GDM visit | 0.556 | 0.037 | 1.075 | | 0.036 |
|  |  | QUICKI at the 1^st^ GDM visit | -35.281 | -68.507 | -2.055 | | 0.038 |
|  |  | HbA1c at the 1^st^ GDM visit (%) | 1.802 | -1.908 | 5.511 | | 0.339 |
|  |  | HbA1c at the last GDM visit (%) | 2.773 | -1.433 | 6.980 | | 0.194 |
|  |  | Triglycerides at the 1^st^ GDM visit (mmol/L) | 1.181 | -0.262 | 2.623 | | 0.108 |
|  |  | HDL at the 1^st^ GDM visit (mmol/L) | -4.455 | -7.325 | -1.586 | | 0.003 |
|  | Fetal Predictors | Cord blood glucose (mmol/L) | -0.604 | -2.524 | 1.315 | | 0.528 |
|  |  | Cord blood insulin (mIU/L) | 0.173 | -0.051 | 0.396 | | 0.127 |
|  |  | Cord blood C-Peptide (μg/L) | 2.092 | -0.924 | 5.107 | | 0.168 |
|  |  | Cord blood HOMA-IR | 0.461 | -0.644 | 1.566 | | 0.404 |
|  |  | Cord blood triglycerides (mmol/L) | -2.322 | -8.651 | 4.007 | | 0.462 |
|  |  | Cord blood HDL (mmol/L) | -2.433 | -12.521 | 7.655 | | 0.628 |

Abbreviations: *OR* Odds Ratio, *CI* Confidence Interval, *BMI* body mass index, *GDM* gestational diabetes mellitus, *BIA* Bioelectrical impedance analysis, *HOMA-IR* Homeostatic Model Assessment for Insulin Resistance, *QUICKI* quantitative insulin-sensitivity check index, *HbA1c* glycated hemoglobin, *HDL* high density lipoprotein, *SD* standard deviation.

^1^ according to the WHO Anthro Survey Analyser tool (42)

Univariate linear and logistic regression analyses at 2 months, adjusted for group allocation (intervention/control) and infant sex and age (where appropriate).

Supplementary table 3. Maternal and fetal predictors of anthropometry at 1 year in univariate regression analysis

| **1 year Anthropometric Parameters** |  | **Predictors** | **β -Coefficient** | **95% CI** | | **p- value** |
| --- | --- | --- | --- | --- | --- | --- |
|  |  |  |  |  |  |  |
| Weight z-score (SD) ^1^ | Maternal Predictors | Prepregnancy BMI (kg/m^2^) | 0.023 | -0.004 | 0.050 | 0.089 |
|  |  | Gestational weight gain (kg) | 0.011 | -0.014 | 0.035 | 0.383 |
|  |  | Weight at the 1^st^ GDM visit (kg) | 0.014 | 0.004 | 0.023 | 0.006 |
|  |  | Fat mass by BIA at the 1^st^ GDM visit (kg) | 0.018 | 0.003 | 0.034 | 0.020 |
|  |  | Glucose at the 1^st^ GDM visit (mmol/L) | 0.185 | -0.119 | 0.489 | 0.231 |
|  |  | Insulin at the 1^st^ GDM visit (mIU/L) | 0.014 | -0.004 | 0.033 | 0.134 |
|  |  | HOMA-IR at the 1^st^ GDM visit | 0.056 | -0.019 | 0.131 | 0.140 |
|  |  | QUICKI at the 1^st^ GDM visit | -3.661 | -8.157 | 0.835 | 0.110 |
|  |  | HbA1c at the 1^st^ GDM visit (%) | 0.633 | 0.152 | 1.115 | 0.010 |
|  |  | HbA1c at the last GDM visit (%) | 0.739 | 0.169 | 1.309 | 0.011 |
|  |  | Triglycerides at the 1^st^ GDM visit (mmol/L) | -0.006 | -0.198 | 0.186 | 0.950 |
|  |  | HDL at the 1^st^ GDM visit (mmol/L) | -0.246 | -0.634 | 0.141 | 0.211 |
|  | Fetal Predictors | Cord blood glucose (mmol/L) | -0.152 | -0.393 | 0.089 | 0.208 |
|  |  | Cord blood insulin (mIU/L) | -0.027 | -0.071 | 0.016 | 0.212 |
|  |  | Cord blood C-Peptide (μg/L) | -0.302 | -0.803 | 0.199 | 0.228 |
|  |  | Cord blood HOMA-IR | -0.195 | -0.443 | 0.054 | 0.121 |
|  |  | Cord blood triglycerides (mmol/L) | 0.321 | -0.554 | 1.196 | 0.462 |
|  |  | Cord blood HDL (mmol/L) | 0.927 | -0.379 | 2.233 | 0.159 |
| BMI (kg/m2) | Maternal Predictors | Prepregnancy BMI (kg/m^2^) | 0.059 | 0.013 | 0.106 | 0.013 |
|  |  | Gestational weight gain (kg) | -0.015 | -0.060 | 0.029 | 0.488 |
|  |  | Weight at the 1^st^ GDM visit (kg) | 0.023 | 0.006 | 0.040 | 0.007 |
|  |  | Fat mass by BIA at the 1^st^ GDM visit (kg) | 0.031 | 0.004 | 0.058 | 0.024 |
|  |  | Glucose at the 1^st^ GDM visit (mmol/L) | 0.292 | -0.239 | 0.824 | 0.279 |
|  |  | Insulin at the 1^st^ GDM visit (mIU/L) | 0.021 | -0.012 | 0.055 | 0.207 |
|  |  | HOMA-IR at the 1^st^ GDM visit | 0.082 | -0.052 | 0.216 | 0.229 |
|  |  | QUICKI at the 1^st^ GDM visit | -4.625 | -12.692 | 3.442 | 0.259 |
|  |  | HbA1c at the 1^st^ GDM visit (%) | 1.002 | 0.154 | 1.850 | 0.021 |
|  |  | HbA1c at the last GDM visit (%) | 1.037 | 0.032 | 2.043 | 0.043 |
|  |  | Triglycerides at the 1^st^ GDM visit (mmol/L) | 0.052 | -0.283 | 0.388 | 0.758 |
|  |  | HDL at the 1^st^ GDM visit (mmol/L) | -0.396 | -1.072 | 0.279 | 0.248 |
|  | Fetal Predictors | Cord blood glucose (mmol/L) | -0.107 | -0.612 | 0.397 | 0.668 |
|  |  | Cord blood insulin (mIU/L) | -0.043 | -0.129 | 0.044 | 0.321 |
|  |  | Cord blood C-Peptide (μg/L) | 0.017 | -0.987 | 1.021 | 0.973 |
|  |  | Cord blood HOMA-IR | -0.261 | -0.764 | 0.242 | 0.299 |
|  |  | Cord blood triglycerides (mmol/L) | -0.483 | -2.021 | 1.054 | 0.528 |
|  |  | Cord blood HDL (mmol/L) | 0.528 | -2.152 | 3.208 | 0.692 |

| BMI z-score (SD) ^1^ | Maternal Predictors | Prepregnancy BMI (kg/m^2^) | 0.043 | 0.012 | 0.074 | 0.006 |
| --- | --- | --- | --- | --- | --- | --- |
|  |  | Gestational weight gain (kg) | -0.012 | -0.041 | 0.016 | 0.400 |
|  |  | Weight at the 1^st^ GDM visit (kg) | 0.017 | 0.006 | 0.028 | 0.003 |
|  |  | Fat mass by BIA at the 1^st^ GDM visit (kg) | 0.023 | 0.005 | 0.041 | 0.011 |
|  |  | Glucose at the 1^st^ GDM visit (mmol/L) | 0.144 | -0.209 | 0.498 | 0.422 |
|  |  | Insulin at the 1^st^ GDM visit (mIU/L) | 0.011 | -0.011 | 0.033 | 0.328 |
|  |  | HOMA-IR at the 1^st^ GDM visit | 0.040 | -0.049 | 0.128 | 0.379 |
|  |  | QUICKI at the 1^st^ GDM visit | -2.220 | -7.540 | 3.099 | 0.411 |
|  |  | HbA1c at the 1^st^ GDM visit (%) | 0.601 | 0.035 | 1.166 | 0.038 |
|  |  | HbA1c at the last GDM visit (%) | 0.645 | -0.002 | 1.292 | 0.051 |
|  |  | Triglycerides at the 1^st^ GDM visit (mmol/L) | 0.045 | -0.177 | 0.266 | 0.692 |
|  |  | HDL at the 1^st^ GDM visit (mmol/L) | -0.318 | -0.765 | 0.130 | 0.162 |
|  | Fetal Predictors | Cord blood glucose (mmol/L) | -0.094 | -0.398 | 0.211 | 0.537 |
|  |  | Cord blood insulin (mIU/L) | -0.050 | -0.102 | 0.002 | 0.060 |
|  |  | Cord blood C-Peptide (μg/L) | -0.436 | -1.058 | 0.186 | 0.163 |
|  |  | Cord blood HOMA-IR | -0.315 | -0.615 | -0.015 | 0.040 |
|  |  | Cord blood triglycerides (mmol/L) | 0.858 | -0.196 | 1.913 | 0.108 |
|  |  | Cord blood HDL (mmol/L) | -0.156 | -1.822 | 1.509 | 0.850 |
| Sum of 4 Skinfolds (mm) | Maternal Predictors | Prepregnancy BMI (kg/m^2^) | 0.061 | -0.265 | 0.387 | 0.712 |
|  |  | Gestational weight gain (kg) | 0.121 | -0.159 | 0.402 | 0.393 |
|  |  | Weight at the 1^st^ GDM visit (kg) | 0.085 | -0.036 | 0.206 | 0.166 |
|  |  | Fat mass by BIA at the 1^st^ GDM visit (kg) | 0.125 | -0.064 | 0.315 | 0.194 |
|  |  | Glucose at the 1^st^ GDM visit (mmol/L) | 1.149 | -2.501 | 4.800 | 0.535 |
|  |  | Insulin at the 1^st^ GDM visit (mIU/L) | -0.019 | -0.265 | 0.227 | 0.880 |
|  |  | HOMA-IR at the 1^st^ GDM visit | 0.035 | -0.947 | 1.017 | 0.944 |
|  |  | QUICKI at the 1^st^ GDM visit | 23.166 | -36.918 | 83.250 | 0.447 |
|  |  | HbA1c at the 1^st^ GDM visit (%) | 6.578 | 0.783 | 12.372 | 0.026 |
|  |  | HbA1c at the last GDM visit (%) | 2.246 | -4.223 | 8.716 | 0.493 |
|  |  | Triglycerides at the 1^st^ GDM visit (mmol/L) | 1.744 | -0.534 | 4.023 | 0.132 |
|  |  | HDL at the 1^st^ GDM visit (mmol/L) | 0.421 | -4.210 | 5.052 | 0.858 |
|  | Fetal Predictors | Cord blood glucose (mmol/L) | 1.008 | -1.617 | 3.633 | 0.440 |
|  |  | Cord blood insulin (mIU/L) | -0.616 | -1.008 | -0.223 | 0.003 |
|  |  | Cord blood C-Peptide (μg/L) | -6.602 | -11.082 | -2.122 | 0.005 |
|  |  | Cord blood HOMA-IR | -3.431 | -5.902 | -0.960 | 0.008 |
|  |  | Cord blood triglycerides (mmol/L) | 4.469 | -4.414 | 13.352 | 0.314 |
|  |  | Cord blood HDL (mmol/L) | -8.630 | -21.911 | 4.651 | 0.195 |

Abbreviations: *OR* Odds Ratio, *CI* Confidence Interval, *BMI* body mass index, *GDM* gestational diabetes mellitus, *BIA* Bioelectrical impedance analysis, *HOMA-IR* Homeostatic Model Assessment for Insulin Resistance, *QUICKI* quantitative insulin-sensitivity check index, *HbA1c* glycated hemoglobin, *HDL* high density lipoprotein, *SD* standard deviation.

^1^ according to the WHO Anthro Survey Analyser tool (42)

Univariate linear and logistic regression analyses at 1 year, adjusted for group allocation (intervention/control) and infant sex and age (where appropriate).
